# Nucleotide excision repair: a versatile and smart toolkit: Nucleotide excision repair

**DOI:** 10.3724/abbs.2022054

**Published:** 2022-05-25

**Authors:** Xiping Zhang, Mengdie Yin, Jinchuan Hu

**Affiliations:** Shanghai Fifth People’s Hospital Fudan University and Shanghai Key Laboratory of Medical Epigenetics International Co-laboratory of Medical Epigenetics and Metabolism (Ministry of Science and Technology) Institutes of Biomedical Sciences Fudan University Shanghai 200032 China

**Keywords:** nucleotide excision repair, transcription-coupled repair, global genome repair, damage recognition, repair mapping

## Abstract

Nucleotide excision repair (NER) is a major pathway to deal with bulky adducts induced by various environmental toxins in all cellular organisms. The two sub-pathways of NER, global genome repair (GGR) and transcription-coupled repair (TCR), differ in the damage recognition modes. In this review, we describe the molecular mechanism of NER in mammalian cells, especially the details of damage recognition steps in both sub-pathways. We also introduce new sequencing methods for genome-wide mapping of NER, as well as recent advances about NER in chromatin by these methods. Finally, the roles of NER factors in repairing oxidative damages and resolving R-loops are discussed.

## Introduction

As the carrier of genetic information, DNA is the target of many endogenous and exogenous genetic toxic agents [1]. The former contain metabolic products such as reactive oxygen species (ROS) and aldehydes [2]; while the latter have a long list including ultraviolet (UV) in the sunlight [3], polycyclic aromatic hydrocarbons from air pollutants [4], aflatoxin from contaminated food [5], chemotherapeutic drugs like cisplatin [6], and natural products such as aristolochic acids [7] and illudin S [8].

These agents cause various types of DNA base modifications and adducts, which will affect base pairing and interfere with DNA replication and transcription [9], and finally threaten genomic stability, resulting in cancer and aging [ 10– 12] . To maintain genomic integrity, DNA repair pathways, mainly base excision repair (BER) and nucleotide excision repair (NER), are evolved to deal with these damages.

The BER pathway utilizes specific glycosylases to recognize and excise the corresponding base modifications, generating apurinic/apyrimidinic (AP) sites which are further processed by APE1 and other BER factors [ 13– 15] . However, there are only 11 glycosylases identified from the human genome [16], and each glycosylase can only recognize a couple of lesions sharing similar structures [17]. Thus, a limited range of damages can be repaired by the BER pathway, while an unpredictable number of structurally heterogeneous base modifications and adducts are left unrepaired. Therefore, a piece of versatile repair machinery is strongly required, and NER is such a pathway. To cope with such a diversity of lesions, NER aims for common features of base modifications and adducts instead of unique structures, which is discussed below.

NER exists in all three domains of life [18], albeit there are two pieces of evolutionarily unrelated machinery: bacterial NER and eukaryotic NER. Intriguingly, NER in bacteria and eukaryotes utilizes the same strategy to recognize various lesions, but the core factors are completely different between the two domains [19]. The bacterial type of NER has been identified in certain species of Archaea in vitro, which is likely due to horizontal gene transfer [20]. In Archaea, some proteins homologous to eukaryotic NER factors were also found [21], but a functional eukaryotic type of NER has not been demonstrated yet [22]. In this review, we will focus on NER in eukaryote, especially in mammalian cells.

Defects of NER factors in humans can cause several inherited diseases with far different phenotypes, including xeroderma pigmentosum (XP), Cockayne syndrome (CS), and ultraviolet sensitive syndrome (UV ^S^S) [23]. Patients with XP are identified by an extremely high chance of skin cancer [24], while CS patients suffer from severe growth retardation, progeria, and photosensitivity but without an increased risk of skin cancer [25]. In contrast, patients of UV ^S^S only have a higher sensitivity to UV in sunlight but have neither developmental abnormality nor elevated possibility of skin cancer [ 26– 28] .

The concept of NER was first raised in the 1960s to describe the “dark repair” of UV damage in *E*. *coli* [29], in contrast to “light repair” operated by photolyases [ 30, 31] . The basic mode of NER, namely “dual incisions”, was revealed in the 1980s for bacteria [ 32– 34] , and in the 1990s for mammalian cells [ 35– 37] , both through *in vitro* reconstituted reactions. In recent years, this mode was finally demonstrated in both eukaryotic and prokaryotic cells [ 34, 38– 40] . Dr. Aziz Sancar is one of the main contributors in this field and was awarded the Nobel Prize in Chemistry in 2015 [41]. Although the mechanism of dual incision reaction was described in considerable details, it is still elusive how repair factors seek and recognize damage efficiently across the genome.

In this review, we will summarize the basic mechanism of NER, and introduce recent advances of the above question by various approaches including biochemistry, cell biology, structure biology, and genomics. We will also discuss the roles of NER factors in repairing some non-classical substrates.

## Molecular Mechanism of Eukaryotic Nucleotide Excision Repair

**Figure 1:**
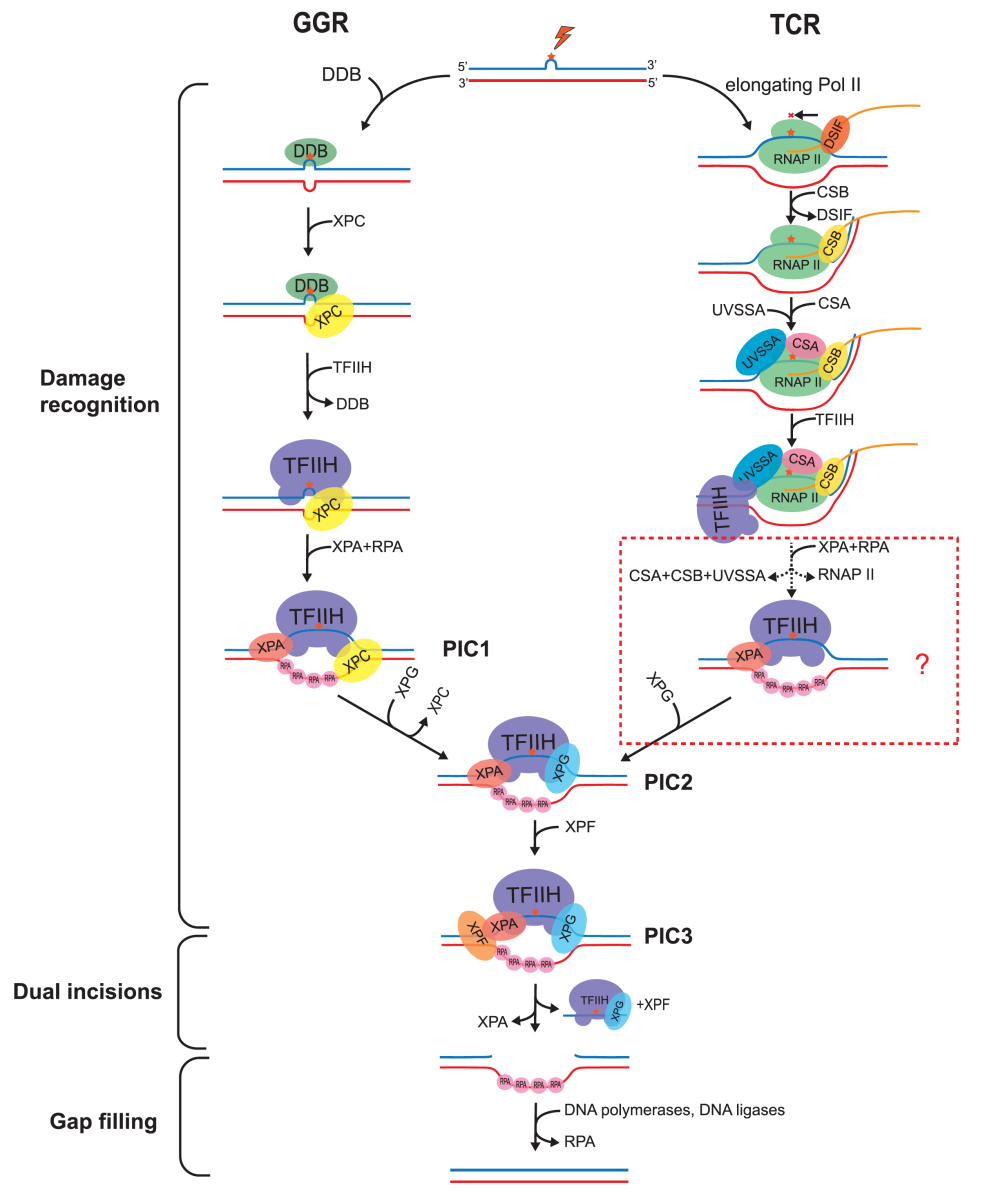
Figure 1 Mechanistic model of nucleotide excision repair In GGR, lesions are first recognized by DDB complex, then XPC, TFIIH, XPA and RPA, XPG, XPF are sequentially recruited while DDB and XPC are released before the dual incisions step. In TCR, blocked Pol II serves as the damage sensor to recruit CSB, CSA and UVSSA, which corporately recruit TFIIH. The details happened between TFIIH loading and dual incisions are unclear. Nonetheless, for both sub-pathways, XPG and XPF make the 3′ and 5′ incisions, respectively, to generate ~26 nt-long ssDNA fragments containing damage which are released in complex with TFIIH and XPG. The resulted gaps are sealed by DNA polymerases and ligases to recover intact dsDNA.

NER removes damaged nucleotides by dual incisions bracketing the lesion and releasing a short (22–30 nucleotides in mammalian cells) single-stranded DNA fragment containing the damage [ 34, 37, 39, 42, 43] . Repair synthesis and ligation are then performed to recover intact double-strand DNA [ 44– 47] . In brief, NER can be divided into 3 major steps: (1) damage recognition, including initial recognition and damage verification; (2) dual incisions and release of excision products; and (3) gap filling, which means repair synthesis and DNA ligation ( Figure 1). In contrast to BER which identifies the specific structures of modified bases, NER recognizes damage-induced double-strand distortions or RNA polymerase II stalled by lesions. The former mechanism deals with damage across the whole genome thus named Global Genome Repair (GGR), while the latter mode only repairs damage on the template strands of transcribed regions, which is called Transcription-Coupled Repair (TCR) [ 48– 50] .

GGR was successfully reconstituted *in vitro* with 6 purified factors (XPC, TFIIH, XPA, RPA, XPF, and XPG) [ 35, 36] , whereas TCR has not been reconstructed till now. Among these 6 factors, XPC is only involved in GGR, and the other 5 factors are necessary for both sub-pathways [ 51– 53] . In addition, the DDB complex is required for GGR initiation *in vivo* [ 54, 55] , while CSB, CSA and UVSSA are needed for TCR [ 26, 28, 56, 57] , as summarized in Table 1.

**Table:** **Table 1** Human nucleotide excision repair factors

Stage	Factor	Component(s)	Enzymatic activity/function	Related disease(s)
Initial damage recognitionfor TCR	CSA	CSA	Ubiquitin E3 ligase (in complex withDDB1, CUL4A & RBX1)	CS or UV ^S^S
	CSB	CSB	Translocase activity	CS or UV ^S^S
	UVSSA	UVSSA	Deubiquitinase (in complex with USP7)	UV ^S^S
Initial damage recognitionfor GGR	DDB	DDB1, DDB2/XPE	Damage recognition in chromatin, ubiquitinE3 ligase (in complex with CUL4A & RBX1)	XP
	XPC	XPC, HR23b, CETN2	Damaged DNA binding	XP
Damage verification andPIC assembly	XPA	XPA	Damaged DNA binding	XP
	RPA	p70, p32, p14	ssDNA binding	–
	TFIIH	p89/XPB, p62, p52, p44,p34, p8, p80/XPD, Cdk7*,Cyclin H*, MAT1*	3′ to 5′ (XPB) and 5′ to 3′ (XPD)helicases	XP or XP-CS
Dual incisions	XPF	XPF, ERCC1	Structure-specific endonuclease for 5′ incision	XP or XP-CS
	XPG	XPG	Structure-specific endonuclease for 3′ incision	XP or XP-CS

* indicates the Cdk-activating kinase complex (CAK) of TFIIH which is not essential for NER. Blue, TCR specific factors; yellow, GGR specific factors; green, common factors for both sub-pathways. CS, Cockayne syndrome; UV ^S^S, Ultraviolet-sensitive syndrome; XP, Xeroderma pigmentosum; XP-CS, Xeroderma pigmentosum-Cockayne syndrome complex (the combination of XP and CS).

### Damage recognition by GGR

The efficiency of GGR is dependent on the extent of double-strand distortion. For instance, UV can induce two major lesions, *i*. *e*., pyrimidine-pyrimidone (6–4) photoproducts [(6–4) PPs] and cyclobutane pyrimidine dimers (CPDs), the former of which causes stronger distortion and is efficiently repaired by GGR [58], while the latter has less impact on DNA structure and is a poor substrate for GGR [59]. Nonetheless, both damages could be excised *in vitro* by the 6-factor system. In this reaction, the lesion is recognized by the cooperation of XPC (usually in the form of XPC-TFIIH), XPA, and RPA. Although either of the three factors is unable to discriminate damages from normal DNA independently, loading of one factor could facilitate binding of other factors, and eventually achieve specific damage recognition [ 19, 60, 61] .

Notably, another GGR-specific factor, the DDB complex, was not required *in vitro*, and the addition of purified DDB did not improve repair efficiency [62]. The DDB complex, composed of DNA damage-binding protein 2 (DDB2/XPE) and DNA damage-binding protein 1 (DDB1), forms a complex with the ubiquitin E3 ligase Cul4A-RBX1 (CRL4 ^DDB2^) [ 63, 64] . Although DDB is dispensable for *in vitro* repair [ 35, 36] , it plays important physiological roles as defects in DDB2/XPE gene strongly impede the repair of CPD *in vivo*, and can also cause xeroderma pigmentosum [ 65– 67] . Therefore, DDB is thought to be involved in the repair of lesions with less distortion ( *e*. *g*., CPDs) in chromatin [ 67, 68] . However, the exact roles of DDB are more complex and not completely clear. Firstly, DDB2/XPE protein has the highest affinity and selectivity to both (6–4) PP and CPD among all GGR factors [ 69, 70] . Local U V irradiation revealed that DDB was recruited to damaged sites ahead of XPC [71]. Furthermore, structure studies suggested the binding of DDB kink the double-stranded DNA and promote the binding of XPC [72]. It was recently reported that DDB can bind to nucleosomal lesions and shift the DNA to expose the lesions facing the nucleosome core [73]. Thus, DDB can help recruit XPC to damage sites, especially those with minor distortion or hard to access. However, it was reported that DDB prefers lesions located in linkers and nucleosome-free regions rather than nucleosome core regions *in vivo* [74]. Consistently, a recently published study indicated that DDB can be recruited to linker regions after UV irradiation to stimulate the displacement of linker histones and relax heterochromatin compaction, and finally facilitate repair in heterochromatin domains [75]. Moreover, upon binding to damage, the CRL4 ^DDB2^ ubiquitin ligase can ubiquitinate surrounding proteins including DDB2 itself, XPC [55], and histones [67], which is thought to promote damage handover [54] and decompaction of damaged nucleosomes [76]. Therefore, DDB can stimulate GGR in both direct and indirect ways.

Unlike the *in vitro* system, XPC is supposed to be the first factor following the recruitment of DDB. XPC itself can bind to DNA and scan for damages both *in vitro* and *in vivo* [77], albeit it cannot efficiently distinguish lesions with weaker DNA distortion from undamaged DNA [78]. Structure studies of Rad4 (yeast homolog of XPC) complex and damaged DNA indicated that it binds to the opposite strand of the lesion and the double-stranded DNA at 3′ downstream to the lesion ( Figure 1) [ 58, 78, 79] . Notably, (6–4) PPs that strongly affect DNA structure can be successfully repaired *in vivo* without DDB though with an apparent delay [80].

Once loaded onto damage sites, XPC can recruit the scaffold factor TFIIH through their interaction [ 81– 83] . TFIIH is a multi-subunit factor involved in both transcription initiation and NER [ 84– 87] . NER requires the TFIIH core complex which consists of 7 subunits, including two DNA helicases (XPB and XPD) and other structural peptides [88], while transcription initiation needs an extra 3-subunit CAK module [89]. As discussed above, initial damage recognition is not a strict process, such that XPC can bind to undamaged DNA or minor base modifications which should not be excised by NER. Therefore, the suitability of NER substrates is verified before dual incisions through cooperative binding and kinetic proofreading, both of which are mediated by TFIIH [ 90– 92] . The loading of TFIIH, together with DDB2 ubiquitination, can stimulate the dissociation of DDB2 and stabilize the binding of XPC [54]. The helicase activity of TFIIH subunits can separate DNA double strands, allowing the binding of XPA and RPA [93]. The structure of the TFIIH-XPA-DNA complex revealed that TFIIH adopt different conformation in transcription and repair, and the binding of XPA can promote and stabilize the conformation change of TFIIH and stimulate the helicase activity of TFIIH on undamaged DNA [ 94, 95] . On the other hand, the presence of bulky adducts ( *e*. *g*., cisplatin-damage), but not minor modifications ( *e*. *g*., AP sites), can reduce helicase activity of TFIIH, and the inhibition is further enhanced by XPA [90]. Therefore, TFIIH is trapped by appropriate bulky adducts with the help of XPA, achieving specific damage verification.

In the *in vitro* reaction, XPC-TFIIH, XPA, and RPA can form a stable complex with damaged DNA, called pre-incision complex 1 (PIC1). Then XPG endonuclease is recruited to damage through its interaction with TFIIH, while XPC leaves the complex, forming pre-incision complex 2 (PIC2). This process is driven by the ATP hydrolysis activity of TFIIH, while XPG can stimulate the helicase activity of TFIIH in the absence of damage. The other endonuclease XPF is finally recruited to assemble pre-incision complex 3 (PIC3) [ 96, 97] . Although XPF has a strong interaction with XPA [98], the loading of XPF also depends on the recruitment (but not the incision) of XPG [99].

### Damage recognition by TCR

RNA polymerase II (Pol II) is efficiently blocked by bulky adducts and serves as a damage sensor to initiate TCR [ 9, 100] . Thus, in comparison with GGR, TCR can only deal with lesions on the template strands of transcribed regions, but it repairs various substrates with similar efficiency despite their different impacts on DNA structure [101], for Pol II indirectly detects bulky lesions by their transcription-blocking feature.

Although TCR was first identified in mouse cells more than 35 years ago [102], it has not been reconstituted *in vitro* till now. Thus, the molecular details of this mechanism are not as clear as GGR. The initial clue came from human genetics that CS was connected with defects in TCR [ 103, 104] , and two genes responsible for CS, *i*. *e*., *CSA* [105] and *CSB* [104], were identified as essential factors of TCR. In 2012, the third factor of TCR, namely UVSSA, was characterized through the study of UV ^S^S [ 26– 28] . Indeed, new TCR players have still been reported even during the last two years [106]. Therefore, the mechanism of TCR is still a hot topic in the DNA repair field.

When an elongating Pol II encounters a lesion and stalls at that site, CSB, a member of the SWI2/SNF2 ATPase family of chromatin remodelers [107], is the first repair factor to be recruited [ 108– 110] . Actually, CSB is required for normal transcription even without bulky adducts [111]. It was reported that Rad26, the yeast ortholog of CSB, can act as an elongation factor to help Pol II to overcome nucleosome barriers *in vitro* [112]. The structure of the Pol II-Rad26 complex revealed that Rad26 binds to DNA upstream of Pol II, and the binding sites of Rad26 overlap with that of the transcription elongation factor Spt4-Spt5 [ 113, 114] . Therefore, it was speculated that when Pol II temporally stalls during elongation, Spt4-Spt5 should be replaced by CSB/Rad26 which can “push” Pol II to overcome “small” barriers. If it is a “large” obstacle like CPD that cannot be bypassed, a stable complex of Pol II-CSB/Rad26-DNA damage will be formed to recruit downstream repair factors, *i*. *e*., CSA and UVSSA [ 111, 115, 116] .

Similar to DDB2, CSA forms a complex with the ubiquitin E3 ligase DDB1-Cul4A-RBX1 (CRL4 ^CSA^) which mediates UV-induced ubiquitination of TCR factors including Pol II, CSB, CSA, and UVSSA, resulting in the instability of this complex [64]. Paused Pol II, CSB, and CSA together recruit UVSSA which is in complex with the deubiquitinase USP7 [56]. CRL4 ^CSA^ and UVSSA-USP7 can cooperatively balance the stability of CSB, as the depletion of UVSSA reduces CSB protein level following UV irradiation. Deficiency of UVSSA will lead to an earlier release of Pol II from damaged sites [117]. However, CSB overexpression cannot rescue the UV hypersensitivity caused by UVSSA mutation, indicating other roles of UVSSA in TCR [28]. It was reported that UVSSA can directly interact with the p62 subunit of TFIIH and is essential for the recruitment of TFIIH [ 56, 81] . Cramer et al. [118] reported the structures of human TCR damage recognition complexes (including Pol II, CSB, CRL4 ^CSA^, UVSSA, *etc*.). In the basic complex, CSB binds to upstream DNA; UVSSA localizes to downstream DNA; CSA sets between them as a bridge. Moreover, their results confirmed that stalled Pol II can induce the replacement of DSIF (Spt4-Spt5) by CSB which “pulls” DNA and facilitates Pol II to move forward. CSA can stimulate the ATPase activity of CSB and help CSB push Pol II. The activity center of CRL4 ^CSA^ contacts Pol II (near K1268, see below) and CSB in two different conformations, while *in vitro* experiments also confirmed that CRL4 ^CSA^ can ubiquitinate Pol II K1268 and CSB.

Despite their essential roles in TCR, mutations in *CSB*, *CSA*, or *UVSSA* genes can cause two different diseases, *i*. *e*., CS or UV ^S^S, respectively, in most cases. However, a few cases of UV ^S^S were reported to be due to defects in CSB [119] and CSA [ 57, 120] , respectively. Although there are several hypotheses about the relationship between genetic defects and phenotypes, the exact underlying mechanism is unclear.

Besides the dedicated TCR factors mentioned above, Pol II can be regarded as another critical damage recognition factor of TCR. It has long been known that RPB1, the catalytic subunit of Pol II, is ubiquitinated following UV irradiation [ 121– 124] . Even so, the UV-induced ubiquitination site of RPB1 was just identified recently [ 125, 126] . Two groups simultaneously reported that K1268 of RPB1 is the main UV-induced ubiquitinated residue, and the K1268 ubiquitination is important for transcription recovery and cell survival after UV treatment [ 125, 126] . However, the roles of K1268 ubiquitination are, to some extent, controversial in two studies. On one hand, Svejstrup and colleagues reported that K1268 ubiquitination mainly regulates the pool of Pol II through UV-induced proteolysis, which is important for DNA damage response and cell survival [126]. On the other hand, Ogi and colleagues found that loss of K1268 ubiquitination impairs the recruitment of TFIIH, thus strongly inhibits TCR [125]. Further studies are required to unveil the roles of K1268 ubiquitination in TCR and transcriptional response to UV damage.

Recently, a general elongation factor Elof1 emerged from independent screens for damage-sensitivity factors [127]. Elof1 is a conserved small protein (~10 kDa) that exists in the Pol II elongation complex [ 128, 129] . The structure of Pol II elongation complexes revealed that Elf1 (yeast orthologue of Elof1) binds to downstream of Pol II on the DNA and plays a role in elongating through nucleosomes [130]. Two back-to-back studies reported that loss of Elof1 greatly impedes the recruitment of UVSSA to damage sites and abrogates TCR [ 106, 131] . The simulated structure suggested that Elof1 binding site on Pol II is close to K1268, and experimental evidence indicated that Elof1 is involved in UV-induced Pol II ubiquitination at K1268. This may explain the mechanistic role of Elof1 in TCR.

Unlike GGR whose mechanism has been well studied, what happens after TFIIH loading remains elusive in TCR. Common NER factors, *i*. *e*., XPA, RPA, XPG, and XPF, should also be recruited to perform dual incisions. However, whether they are recruited in the same way as in GGR or not is unknown. Furthermore, the fate of TCR-specific factors including Pol II, CSB, CSA, and UVSSA is an open question. Although *in vitro* experiments indicated that paused Pol II would not inhibit dual incision reaction by GGR [116], it was supposed that Pol II stalling at damage sites should either be backtracked or removed during TCR [ 123, 132] . Evidence from recent sequencing-based studies suggested that stalled Pol II should dissociate from damage sites, since nascent transcriptions mainly restart from transcription starting sites after UV irradiation [ 133– 136] .

### Dual incisions and release of excision products

*In vitro* studies indicated that after the assemble of PIC3, two structure-specific endonucleases, XPG and XPF, sequentially carry out incisions on the damaged strand at 4–7 nucleotides downstream and 16–21 nucleotides upstream to the lesion, respectively [ 37, 99] . The primary excised oligomers are released in complex with TFIIH, and then slowly degraded to shorter fragments 15-20-nt in length which are bound by RPA [137]. However, it was not clear whether *in vivo* repair has the same excision pattern, especially in TCR that cannot be reconstructed *in vitro*. This question was resolved by the detection of *in vivo* excised oligomers from UV-irradiated human cells [101]. Analyses of *in vivo* excision products suggested that both nucleases make incisions at the same positions as *in vitro* reaction, generating excision products of the same length and bound by the same proteins, *i*. *e*., TFIIH and XPG. The *in vivo* degradation rate of primary products is faster than *in vitro*, while the 15–20-nt long degraded products are also bound by RPA, and further degraded to fragments that are too short to be detected [101]. However, the nucleases responsible for this degradation process are not clear. More importantly, excision products from XP-C cells which have only TCR show identical properties with those from CS-B cells that have only GGR, indicating that dual incisions and release of excision products are the same for both GGR and TCR *in vivo* [101]. In addition to human cells and UV damage, the *in vivo* excision products were identified in different species including lemur cells [138], mice [ 139– 141] , *Drosophila* [142], *Arabidopsis* [ 39, 143, 144] , and yeast [145], and for various damage types such as cisplatin [ 139– 141, 146] and BPDE [147], suggesting that “dual incisions” is a universal mechanism for eukaryotes.

### Repair synthesis and ligation

In most cases, the excision gaps are directly filled by DNA polymerase ε in proliferating cells or polymerase δ/κ in non-proliferating cells in the presence of proliferating cell nuclear antigen (PCNA) [ 40, 45, 148] . The size of repair patches is about 30 nt [ 149, 150] , consistent with the length of excised fragments. However, a small portion of excision gaps are enlarged by exonuclease I (Exo I) to generate a long stretch of ssDNA which is occupied by RPA and serves as the initial signal for ATR-mediated DNA damage response [ 151– 153] . In dividing cells, the final nick is mainly sealed by DNA ligase I [ 40, 45] , while the XRCC1-ligase3 complex performs ligation in non-dividing cells. Although the gap-filling process is not essential for dual incisions, inhibition of repair synthesis and ligation can hinder the degradation of RPA-bound fragments and reduce the repair rate of UV damages [154].

### Genome-wide Maps of Nucleotide Excision Repair

**Figure 2:**
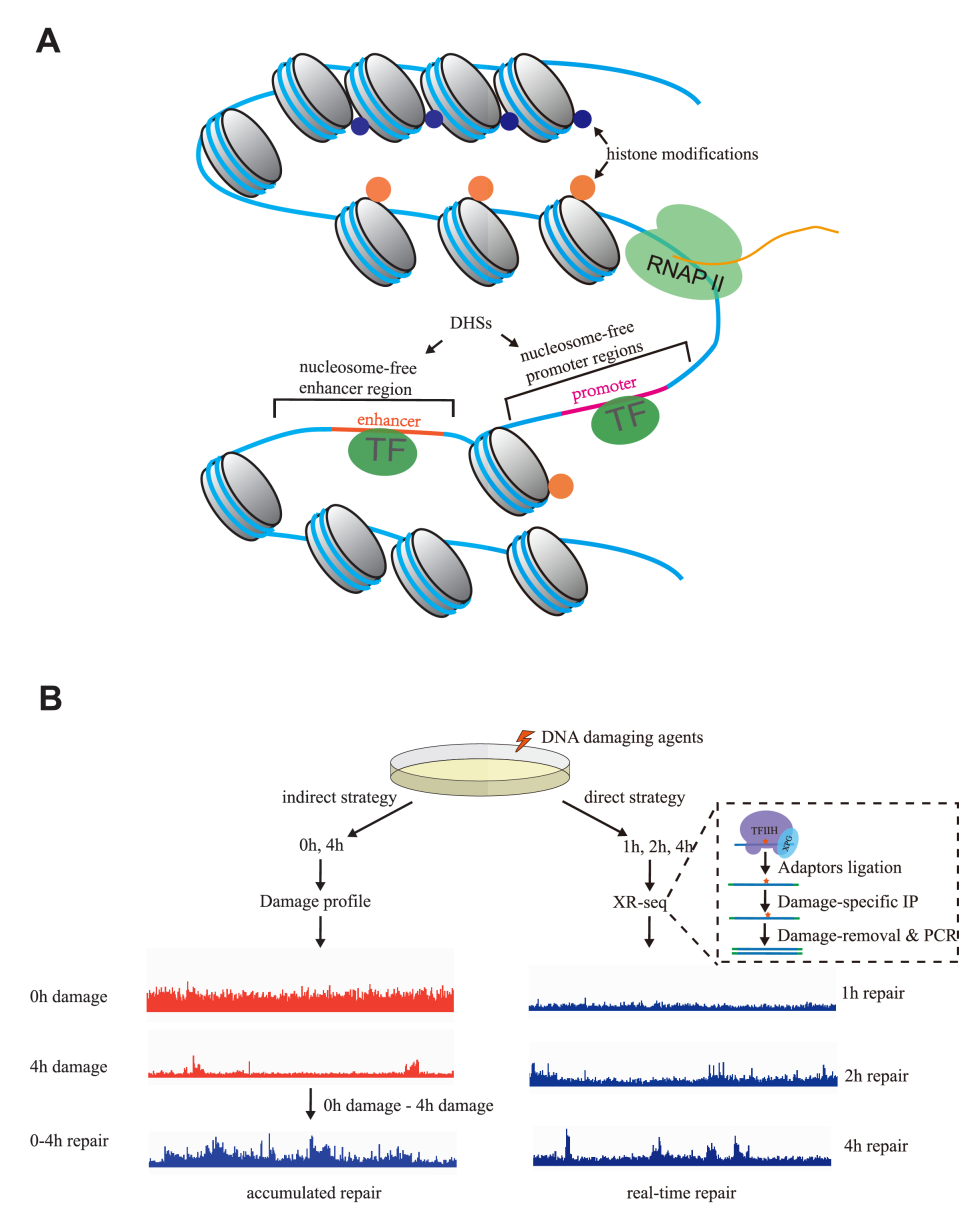
Figure 2 Nucleotide excision repair in chromatin environment (A) Chromatin factors that may affect NER, including histone modifications, transcription, transcription factor binding, nucleosome positioning and genome accessibility. TF is short for transcription factor. (B) The methodology to map NER across the genome. The indirect strategy measures genomic profiles of damage at different time points and acquires the pattern of accumulated repair during this time course by comparing damage profiles. The direct strategy (XR-seq) maps NER by capturing and sequencing excised oligonucleotides and obtains the snapshot of repair at a specific time point.

In eukaryotic cells, genomic DNA is packaged with histones. Thus, NER is performed in chromatin rather than on naked DNA [155]. Meanwhile, complicated events occurring in chromatin, including transcription and DNA replication, also have impacts on NER ( Figure 2A). In order to investigate how chromatin environment affects NER, many efforts have been made to acquire genome-wide maps of NER and unveil the correlations between NER and chromatin compaction, transcription, *etc*.

### Methodology for mapping nucleotide excision repair

There are two strategies to profile NER across the whole genome ( Figure 2B). The first one is achieved by assessing the genome-wide distribution of bulky adducts (the substrates of NER) in a time course and calculating the rate of disappearance at different loci throughout the genome. Accordingly, a couple of methods were developed in recent years to map adducts at base resolution. One type of these methods, including CPD-seq [156], Adduct-seq [157], and Circle-damage-seq [158], took advantage of T4 Endonuclease V to cut the damaged DNA strand at CPD sites and captured these DNA ends for sequencing. Other methods such as Damage-seq [ 146, 159] and cisplatin-seq [160] utilized specific antibodies [for CPD, (6–4) PP, cisplatin adducts, *etc*.] or a damage-binding protein (engineered HMGB1 for cisplatin adducts) to capture DNA strands containing lesions, respectively, and then detected the exact positions by high-fidelity DNA polymerases which can be blocked by the lesions. However, it is not a good choice to measure repair by comparing damage distribution in a time course, especially when only a small portion of damage is repaired, *e*. *g*., at early time points or in cells partially deficient in repair, since it would be inaccurate to determine a small value by subtracting a big number from another big number.

The second strategy is directly profiling NER by isolating and sequencing *in vivo* excision products. The method, named XR-seq (eXcision-Repair sequencing), captured primary excision products by co-immunoprecipitation with anti-TFIIH (XPB or p62) or anti-XPG antibodies, and then added adaptors to both ends of excision products. Afterwards ligation products were purified by immunoprecipitation with damage-specific antibodies, and the lesions were directly reversed by photolyases (for UV-induced damages) or chemical treatment (for cisplatin-adduct) to enable PCR-amplification and sequencing [ 146, 161, 162] . Comparing with indirect assays that assess repair by subtraction, XR-seq possesses much higher sensitivity and provides snapshots of repair instead of cumulative changes of damage. XR-seq can detect repair from 1 min to 48 h after UV irradiation [163], and profile GGR and TCR in TCR-deficient and GGR-deficient cells, respectively [162]. Notably, the genome-wide distribution of damage is also valuable for the exploration of repair, as the repair events captured by XR-seq are determined by both relative repair capacity and local damage density, and the cumulative repair maps are complementary to the snapshots.

### Patterns of nucleotide excision repair throughout the genome

The repair maps by XR-seq revealed intriguing patterns of TCR and GGR. In XPC mutant cells that have only TCR, repair of both UV-induced damages occurs exclusively on the template strands of transcribed regions. Clear repair signals on the coding strands at the upstream regions of TSSs and on both strands around enhancers suggested that the bidirectional transcription by Pol II in mammalian cells is also able to trigger TCR [162]. In contrast, rDNA regions transcribed by Pol I showed no preferential repair of templates strands, indicating that Pol I cannot cause TCR [164].

In addition to TCR, GGR is also promoted by transcription. In CSB mutant cells that lack TCR, elevated repairs on both strands around active TSSs were observed, probably due to the relaxed chromatin within these regions [162]. Furthermore, GGR is also related to other factors including histone modifications, DNase I hypersensitive sites (DHSs), super-enhancers, nucleosome occupation and transcription factor binding [ 163, 165– 168] . In general, “open” regions with active transcription and high accessibility are more accessible for the repair factors and thus repaired faster.

However, in the TSS surrounding regions where damages are repaired faster in general, the impaired repair was observed at specific loci. For instance, time-course XR-seq identified a valley at early time points at downstream region (less than 1kb from TSSs) which turned to be a peak at late time points [165]. The location of the valley and peak coincided with the H3K4me3 peak, which is thought to reflect the first nucleosome downstream of TSSs [169]. Damage-seq verified that the repressed repair at early time points resulted in the accumulation of damage at these loci, which caused the late repair peak [165]. Hindered repair was also observed at transcription factor binding sites in the upstream regions of TSSs, which is related to increased mutation frequencies in cancer genomes [ 167, 168] . This phenomenon was attributed to transcription factor binding which inhibited the access of repair factors. The heterogeneity of repair was generally more obvious at early time points, e.g. repair hotspots were identified at super-enhancers as early as 1 min, and disappeared with time, likely due to the change of damage distribution (as described above) and UV-induced alteration of chromatin compaction [163].

An interesting question is the contributions of TCR and GGR in the repair of different damages, *e*. *g*., UV-induced (6–4) PP and CPD. In mutant cell lines which have only one sub-pathway of NER, both lesions have similar repair patterns. In repair-proficient cells, (6–4) PP repair showed virtually no strand bias, like that in TCR-deficient cells, indicating that this lesion is mainly eliminated by GGR [162]. On the other hand, CPDs on template strands are preferentially repaired, although repair signals on the non-template strands can also be detected [162]. As discussed above, (6–4) PP induces stronger double-strand distortion than CPD, thus is more readily to be eliminated by GGR. The strand difference of CPD repair decreases over time, due to the disappearance of damages on template strands [159]. Surprisingly, at a very early time point (12 min), no asymmetric repair of CPD on two strands was observed in NHF1 human fibroblasts and HeLa cells, implying a delay of TCR after UV [ 163, and our unpublished data]. This phenomenon could only be detected by direct measurement of repair ( *e*. *g*., XR-seq), and the underlying mechanism is unknown.

XR-seq was also used to detect the repair of cisplatin-induced damage in mice [ 139– 141] . Repair maps at different time points of one day revealed the impact of circadian rhythm in two ways. Firstly, the template strands of circadian-controlled genes are preferably repaired when they are being actively transcribed, which is driven by TCR. In contrast, the repair of non-template of all genes and intergenic regions peaks at Zeitgeber time ZT08 when the expression of XPA gene is upregulated by circadian rhythm, indicating the influence of circadian on GGR [ 139, 140] . Moreover, repair in different organs (kidney, liver, lung, and spleen) of mice are shown to be related to tissue-specific transcription patterns and epigenomic profiles [141]. Therefore, NER in living animals is much more complicated and regulated by many factors not existing in cultured cells.

### The Roles of NER Proteins other than Bulky Adducts Repair

It is well known that TFIIH is an essential factor for transcription initiation [170], while RPA is involved in many DNA-related events like replication [171]. The rest of NER factors also possess other functions, since they are not restricted to specific lesions. The recognition factors identify damage by the double-strand distortion or blocked Pol II, no matter they are proper substrates of NER or not. The nucleases, XPF and XPG, just recognize DNA with flap-structure and cut at the single strand-double strand junctions [172]. Therefore, they can operate on DNA with similar property or structure, and perform other functions. Indeed, XPF is also an essential factor in the Fanconi Anemia pathway for the repair of inter-strand crosslink damage [173]. Here we will discuss the roles of NER factors in the repair of oxidative damage, and the important physiological functions of the NER nucleases in resolving R-loops.

### NER proteins and oxidative DNA damage

In general, oxidative damage is eliminated by BER in mammalian cells [174]. However, some “bulky” oxidative lesions, *e*. *g*., 8,5′-cyclo-2′-deoxyadenosine and 8,5′-cyclo-2′-deoxyguanosine, are thought to be repaired by NER [175]. In addition, further oxidation products of the most common oxidative lesion 7,8-dihydro-8-oxo-2′-deoxyguanosine (8-oxo-dG), *i*. *e*., spiroiminodihydantoin and guanidinohydantoin, were found to be excised by both BER and NER *in vitro* by cell extracts that have both BER and NER systems, while 8-oxo-dG was preferred to be repaired by BER under the same condition [ 175, 176] . Although NER excision products cannot be detected under that condition, both 8-oxo-dG and its repair intermediate abasic sites can be recognized by *in vitro* reconstituted NER system [177], albeit they are efficiently removed by OGG1 and APE1 via BER pathway *in vivo* [178]. Whether NER can repair these lesions in the absence of BER *in vivo* is unclear.

Besides the potential involvement of the whole NER pathway, individual repair factors may participate in the repair of oxidative damage in collaboration with BER pathway, as reviewed by Kumar *et al*. [16]. Among NER factors, XPA, XPG, CSA, CSB, UVSSA, XPC, and DDB were all reported to stimulate the repair of oxidative damage like 8-oxo-dG in different studies [ 175, 179– 181] . However, the conclusions are to some extent conflicting. For instance, XPC was reported to be able to stimulate the activity of OGG1 directly [182]. However, genetic experiments indicated that XPC and XPA are involved in the same 8-oxo-dG repair pathway which may be different from that of CSB and OGG1 [181]. The roles of XPG and XPA in oxidative damage repair are also discordant [ 177, 183, 184] . Meanwhile, Guo *et al*. [185] identified transcription-coupled repair of 8-oxo-dG in a CSB-dependent manner, and the recruitment of CSB to oxidative damage sites was also verified by other studies [ 181, 186, 187] . However, as 8-oxo-dG is unable to block Pol II [188], the underlying mechanism is unknown. Finally, a recent study reported the role of DDB in BER [189]. The existence of nucleosomes can greatly inhibit the activity of DNA glycosylates, while DDB was shown to play a role in repairing nucleosomal oxidative damages, just as it did in NER [189]. Further work is needed to clarify the functions and underlying mechanisms of NER factors in the repair of oxidative damage.

### The roles of NER nucleases in resolving R-loops

R-loop is a specific 3-strand structure consisting of a DNA-RNA hybrid and displaced single-stranded DNA [190]. It can be physiologically formed during transcription and is involved in multiple cellular processes, including transcription regulation and termination [191], class switch recombination of immunoglobulin genes [ 192, 193] , etc. However, R-loop can also be induced accidentally and cause genome instability. The flap structure of R-loop makes it a potential substrate of the two NER endonucleases XPG and XPF [194]. It was reported that the absence of the RNA/DNA helicases Aquarius causes the accumulation of R-loops which are further digested by XPG and XPF to generate DSBs. This process depends on the TCR factor CSB and common NER factors TFIIH and XPA, thus is thought to be a TCR-like reaction [194]. However, since R-loops are behind the elongating RNA polymerases, how they can trigger a TCR-like reaction is unclear.

Another study reported that R-loops can stimulate high-fidelity DSB repair by a Rad52 and XPG-dependent mechanism [195]. DSBs in actively transcribing regions can induce R-loops which help recruit Rad52 to facilitate the high-fidelity homologous recombination repair (HR) and suppress the error-prone non-homologous end-joining (NHEJ). In this process, XPG but not XPF is recruited by Rad52 to resolve R-loops and initiate homologous recombination repair. This study revealed the role of XPG in DSB repair via its activity on R-loops.

A more recent study revealed another mechanism for XPG and XPF to be enrolled in resolving R-loops [196]. When R-loops are induced by RNA polymerase stalling, *e*. *g*., in the case of transcription-blocking damage, the splicing factor XAB2 can interact with XPG and XPF-ERCC1 independent of other NER factors to stimulate the processing of R-loops and play a role in maintaining genome integrity. These studies suggested that the NER nucleases, especially XPG, are involved in R-loop processing in multiple ways.

## Conclusions and Perspectives

Although the basic mechanism of NER was unveiled more than 20 years ago, the molecular details of TCR, as well as that of the initial damage recognition by DDB in GGR, remained unclear for a long time due to the lack of the *in vitro* system. Significant progress has been made in the past few years based on the advancement of methodologies in structural biology, *in vivo* imaging, genomics, high-throughput screen, *etc*. However, there are still a couple of remaining questions: (1) Does DDB play other roles in GGR in addition to its reported functions? How do different functions of DDB coordinate in response to UV? (2) How do local chromatin compaction and histone modifications change following UV irradiation across the genome? How do they affect GGR? (3) What determines the phenotype of TCR-deficient patients, *i*. *e*., CS or UV ^S^S? And why do patients possessing some TFIIH, XPF and XPG mutations have CS-like phenotypes? (4) What are the endogenous substrates of NER? Are they related to CS, especially neuro-associated phenotypes? (5) How is a lesion transferred from damage recognition factors to the pre-incision complex during TCR? What is the fate of damage-blocked Pol II and associated TCR factors? (6) Does NER serve as a backup of BER in oxidative damage repair? How important are NER factors for the repair of oxidative damage? (7) Does XPG participate in the processing of all R-loops? Is there any general mechanism for XPG (and XPF) to involve in R-loop resolving?

New answers to the above questions will certainly emerge in next few years, which can help to reveal the molecular details underlying NER and uncover the link between NER and human health diseases.
